# Persistent X-ray-activated phosphors: mechanisms and applications

**DOI:** 10.1038/s41377-022-00808-6

**Published:** 2022-05-04

**Authors:** Cyrille Richard, Bruno Viana

**Affiliations:** 1 Université Paris Cité, CNRS, INSERM, UTCBS, Unité de Technologies Chimiques et Biologiques pour la Santé, Faculté de Pharmacie, 75006 Paris, France; 2 IRCP, CNRS UMR8247, PSL University, Chimie ParisTech, 11 Rue P&M Curie, 75005 Paris, France

**Keywords:** Optics and photonics, Lasers, LEDs and light sources

## Abstract

Trivalent lanthanides in wide bandgap fluoride or phosphate hosts can present persistent luminescence between 200 nm and 1.7 µm after charging by X-rays. Mechanisms are reviewed and applications envisioned.

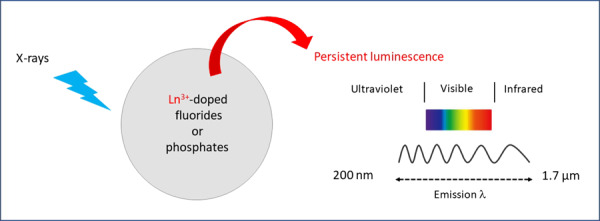

Persistent luminescence is controlled by a slow liberation of trapped charge carriers at body temperature by a simple thermal de-excitation process. It can last for a few minutes to hours after the removal of the excitation source. The persistent luminescence mechanisms can be envisioned from Physics or Chemistry points of view. With material Chemistry vision, two main strategies are envisioned for the trapping process which is the initial and very important step: first, the intrinsic defects and the optimization of the trapping by stoichiometric variation and thermal annealing, and second, a trap optimization by co-doping. This latter strategy has been intensively tested to enhance the persistent luminescence, most of the time by co-doping with one or two lanthanides cations. Following these two distinctive approaches, the physics behind the persistent luminescence mechanisms is not simple and several different models have been proposed during the past years^1–6^. A *global model* in which the transfer of the trapped/detrapped charges occurs through the conduction or valence bands, or with both^7^. In that case, block functions of the carriers are delocalized and indeed this model can be named a global model^8^. Materials with intermediate bandgap values ranging between 3 and 7 eV such as sulfide, oxysulfide, and oxides, namely, garnets, gallate, phosphates, aluminates., etc. required ultraviolet (UV)/blue light for efficient charging through the bandgap and in that case, the so-called bandgap engineering could generally be applied^1,7,9^. On the contrary, a *local model* considers a direct electron transfer between activator and trapping centers^10,11^. Arguments for the two models are now well established and the energy required for the charging process, for instance, could control the process: high energy for global model while low energy is associated with local defect such as antisites, for instance, that are well established in garnets^12–15^, perovskite^16^, and spinel materials^17,18^. Calculations as proposed in ref. ^19^ further validate the so-called *local model*.

Very recently, X-rays have been used as charging light in various materials. In materials that could also be charged by UV or/and visible light such as ZnGa_2_O_4_:Cr, but also in wide bandgap materials which cannot be charged by usual lamps due to their large bandgap (>12 eV)^20^. These materials are mainly fluorides such as NaLnF_4_ (Ln = Lu, Y, Gd, La) with bandgap values in the range 12–14 eV^21,22^. In that case, *local trapping and detrapping model* could well explain the observed persistent luminescence. Li et al. described the local defects in wide bandgap materials such as in NaYF_4_:Ln^3+^-doped materials with anions vacancies^22^ created under X-ray charging. Notice that the efficiency of such charging can be very high in these nanomaterials as measured in Na(Gd,Lu)F_4_:Tb^3+^ to be 2.8 × 10^16^ photons/g^23^, which is surprisingly high for nano-sized particles and comparable to the commercial SrAl_2_O_4_:Eu^2+^,Dy^3+^ bulk persistent phosphor where the *global model* is the most relevant (de)trapping model. Another remarkable example of local defect is presented in Nd, Ho, Tm, and Er:NaYF_4_@NaYF_4_ core–shell nanoparticles in ref. ^24^. Fluoride vacancies and/or fluoride Frenkel defects (vacancy–interstitial pairs) could be formed (see Fig. 2 in ref. ^24^). Some controversy still remains for these wide-bandgap hosts as in another recent work, Zhuang et al.^25^ explain (see Fig. 2a, b in ref. ^25^) that the persistent luminescence mechanism occurs by *global model* but notice that there is some discrepancy in the bandgap value of NaYF_4_ fluoride host, 13 eV reported in ref. ^21^ (see SI7 in ref. ^21^) in regard to 12 eV in^25^, while charge carriers are not easily delocalized in these compounds. Furthermore, as reported by Li et al.^22^, *global model* cannot well explain the persistent luminescence for the fluoride hosts doped with Gd^3+^, then efforts are still required to better model this amazing phenomenon taking into account either a Physics or material Chemistry point of view.

Through an appropriate selection of the Ln^3+^ element in the synthesis of fluoride or phosphate hosts, a wide range of persistent luminescence emission, from 200 nm up to 1.7 µm, can be obtained after X-ray excitation (30 mA, 40 kV). Since the photon counts are much higher when materials are exposed to X-rays when compared to another light source, persistent phosphors developed by Li et al. should have many applications^22^ among those listed in Fig. 1.

**Fig. 1:**
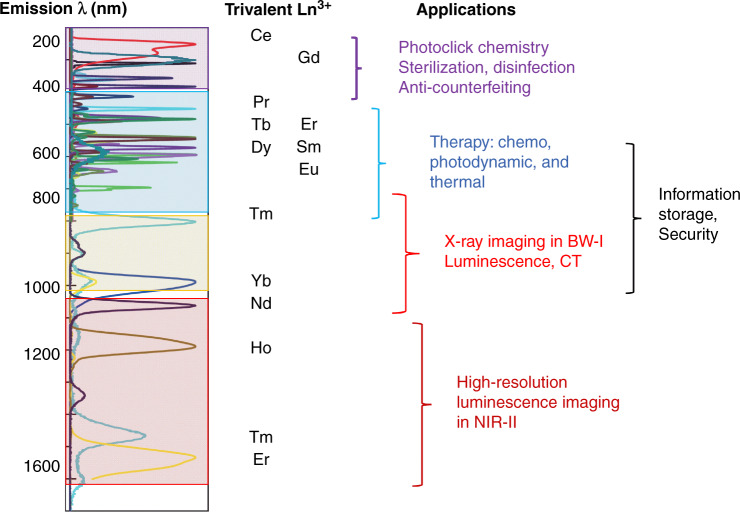
Emission wavelength of Ln^3+^-doped persistent fluorides or phosphates after X-ray excitation and possible applications. Adapted from the original manuscript ref. ^22^

UV light irradiation in the 200–400 nm region may pose considerable phototoxicity to living cells. One way to solve this problem is to use materials that are excited at smaller wavelengths while emitting deep UV. Ce^3+^- or Gd^3+^-doped NaYF_4_ have the capability of UV emission at 250 or 300 nm after being excited by X-rays. Such phosphors are likely to find applications, for example, in photoclick chemistry for which high energy over a long time is needed to create covalent bonds^26^. But other applications for which high energy is required could also be envisioned, such as sterilization and disinfection^27^.

When moving to the visible range, such presented X-ray-activated phosphors should also find applications either in vitro for the development of biosensors^28^ or in vivo for imaging^29^ as well as in therapy^30^. Bioimaging applications can be improved when using probes emitting in BW-I (650–980 nm) or with nanoparticles emitting in BW-II (1000–1600 nm) since this allows imaging of deeper tissues and it gives access to images with better spatial resolution^24^. Here again, the luminescent phosphors developed by Li et al. could be particularly useful. In addition to these applications in biology, many more applications of luminescent phosphors excited by X-rays can be envisioned not only for anti-counterfeiting, information storage, and security^25^ but also in cryopreservation and photocatalysis^31^.
